# Supplementary material for: Health education improves referral compliance of persons with probable Diabetic Retinopathy: A randomized controlled trial
Source: PLoS One. 2020 Nov 12;15(11):e0242047. doi: 10.1371/journal.pone.0242047 (PMC7660573; doi:10.1371/journal.pone.0242047)
Supplement: S1 Table — (DOCX) [file pone.0242047.s001.docx]

| Variables | N (%) |
| --- | --- |
| Sex |  |
| Women | 154 (51.5) |
| Men | 145 (48.5) |
| Experiences disability as a result of vision problem |  |
| Strongly agree  Agree  Neutral  Disagree  Strongly disagree | 5 (1.7)  26 (8.7)  5 (1.7)  263 (88.0)  0 (0) |
| Requires someone else’s assistance to perform day to day activities as a result of vision problem | |
| Strongly agree  Agree  Neutral  Disagree  Strongly disagree | 1 (0.3)  23 (7.7)  4 (1.3)  269 (90.0)  2 (0.7) |
