# Supplementary material for: Health education improves referral compliance of persons with probable Diabetic Retinopathy: A randomized controlled trial
Source: PLoS One. 2020 Nov 12;15(11):e0242047. doi: 10.1371/journal.pone.0242047 (PMC7660573; doi:10.1371/journal.pone.0242047)
Supplement: S2 Table — (DOCX) [file pone.0242047.s002.docx]

| Intervention type | | | Primary Endpoint  (Returned Yes/No) | | Total |
| --- | --- | --- | --- | --- | --- |
|  |  |  | Returned | Did not return |  |
| Standard Care | does patient need someone else's assistance to perform day to day activities, as a result of vision problem | Agree | **3** | 13 | 16 |
|  |  | Neutral | 0 | 3 | 3 |
|  |  | Disagree | 40 | 95 | 135 |
|  |  | Strongly disagree | 1 | 1 | 2 |
|  | Total | | 44 | 112 | 156 |
| Health Education | does patient need someone else's assistance to perform day to day activities, as a result of vision problem | Strongly agree | 0 | 1 | 1 |
|  |  | Agree | **6** | 1 | 7 |
|  |  | Neutral | 1 | 0 | 1 |
|  |  | Disagree | 85 | 49 | 134 |
|  | Total | | 92 | 51 | 143 |
| Total | does patient need someone else's assistance to perform day to day activities, as a result of vision problem | Strongly agree | 0 | 1 | **1** |
|  |  | Agree | **9** | 14 | **23** |
|  |  | Neutral | 1 | 3 | 4 |
|  |  | Disagree | 125 | 144 | 269 |
|  |  | Strongly disagree | 1 | 1 | 2 |
|  | Total | | 136 | 163 | 299 |
