# Supplementary material for: Health education improves referral compliance of persons with probable Diabetic Retinopathy: A randomized controlled trial
Source: PLoS One. 2020 Nov 12;15(11):e0242047. doi: 10.1371/journal.pone.0242047 (PMC7660573; doi:10.1371/journal.pone.0242047)
Supplement: S1 File — (PDF) [file pone.0242047.s003.pdf]

(ANNEXURE D)

Informed Consent to Participate in a Research Study

(only fill out for adults; children are not included in survey)

Duplicate copy of Informed Consent shall be provided to participant

**Title of Study:**	Integrating Diabetic Retinopathy into mainstream Public Health in Bangladesh		
**Name of Investigator/ Enumerator:**		**Phone:**	

Introduction

- You are being asked to be in a research study.
- You were selected as a possible participant because as a registered patient with diabetes at the Barisal DAB center, you have been referred to the eye care unit at Barisal Medical College and Hospital (BMCH) to undergo high-resolution fundus photography. However, it seems that you could not make time to visit the Eye Consultant there. Since your appointment with the Eye Consultant is still pending, we would like to talk to you to get your insights on how to strengthen the existing referral system to be more effective so that you will be empowered to attend your appointment.
- If you permit me, I can read out the consent form to you in Bengali or you may want to read it as well. Please feel free to ask any questions that you may have before agreeing to be in the study.

Purpose of Study

- The purpose of the study is come up with recommendations to strengthen the existing referral pathway from DAB to BMCH (and vice versa).
- This is because at the moment, we have seen that just like yourself, some registered patient with diabetes at the Barisal DAB center, who are being referred to the eye care unit at Barisal Medical College and Hospital (BMCH) to undergo high-resolution fundus photography are missing their appointments.
- Ultimately, this research may be published as part of a book, presented as a paper at conferences, or as part of a journal.

Description of the Study Procedures

- If you agree to be in this study, you will be asked to answer questions about your demographic characteristics, knowledge and awareness about diabetes and management of any vision problems, information you received from DAB center about management of any vision problems, your understanding about the existing referral pathway.
- It will take about 40 minutes for this interview to complete.

Risks/Discomforts of Being in this Study

- There are no reasonable foreseeable (or expected) risks as there will be no invasive medical interventions.

Benefits of Being in the Study

- The information you provide and the perspectives you shine during the course of this interview will be used by investigator to provide recommendations to policy makers and stakeholders to develop and strengthen the eye care referral system for patients with diabetes. Thus this interview will also benefit the broader community and the public health sector.

Confidentiality

- This study is anonymous. We will not be collecting and retaining any information that will make it possible to identity you.
- The records of this study will be kept strictly confidential. Research records will be kept in a locked file, and all electronic information will be coded and secured using a password protected file. We will not include any information in any report we may publish that would make it possible to identify you.
- Your identity will not be disclosed in the material that is published.

Payments

- You will not receive any monetary benefits or payments as reimbursement for the valuable time you provide during the course of this interview. Your contribution in terms of time and the information you provide will have to be completely voluntary, which may benefit you and your wider community.

Right to Refuse or Withdraw

- The decision to participate in this study is entirely up to you. You may refuse to take part in the study *at any time* without affecting your relationship with the investigators of this study. Your decision will not result in any loss or benefits to which you are otherwise entitled. You have the right not to answer any single question, as well as to withdraw completely from the interview at any point during the process; additionally, you have the right to request that the interviewer not use any of your interview material.

Consent

- Your signature/thumb impression below indicates that you have decided to volunteer as a research participant for this study, and that you have read and understood the information provided above.

Respondent's Name:

Respondent's Signature: Date:

Investigator's Name:

Investigator's Signature: Date:

Witness Name:

Witness Signature: Date:
