# Supplementary material for: Health education improves referral compliance of persons with probable Diabetic Retinopathy: A randomized controlled trial
Source: PLoS One. 2020 Nov 12;15(11):e0242047. doi: 10.1371/journal.pone.0242047 (PMC7660573; doi:10.1371/journal.pone.0242047)
Supplement: S2 File — (PDF) [file pone.0242047.s004.pdf]

অবহিতক্রমে সম্মতি পত্র

একটি গবেষণা অধ্যয়ন অংশগ্রহণের সম্মতি (শুধুমাত্র প্রাপ্তবয়স্কদের জন্য; শিশুদের জরিপের মধ্যে অন্তর্ভুক্ত করা হয় না)| অংশগ্রহণকারীকে জ্ঞাত সম্মতির অনুলিপি প্রদান করা হবে|

**অধ্যয়ন শিরোনাম:**	বাংলাদেশের মূলধারার জনস্বাস্থ্যের ক্ষেত্রে ডায়াবেটিক রিটিনোপ্যাথি সংহত করা		
**তদন্তকারীর নাম:**		ফোন	

ভূমিকা

- আপনাকে একটি গবেষণা অধ্যয়ন হতে জিজ্ঞসা করা হচ্ছে
- আপনাকে সম্ভাব্য অংশগ্রহণকারী হিসেবে নির্বাচিত করা হয়েছে কারণ বরিশাল ড্যাব সেন্টারের নিবন্ধিত ডায়াবেটিসের রোগী হিসাবে আপনাকে বরিশাল মেডিক্যাল কলেজ ও হাসপাতালের চক্ষু সেবা ইউনিটে যেতে বলা হয়েছে| তবে মনে হচ্ছে আপনি এই খানে আই কনসালটেন্ট দেখার জন্য সময় করতে পারেন নাই| যার কারনে কনসালটেন্টের সাথে আপনার এ্যাপয়েন্টমেন্ট এখনো মুলতুবী আছে| তাই আমরা বর্তমান রেফারেল পদ্ধতি কিভাবে আরো কার্যকর ও শক্তিশালী করা যায় সেটা নিয়ে আপনার সাথে কথা বলতে চাই|
- যদি আপনি আমাকে অনমুতি দেন, আমি আপনার কাছে সম্মতিপত্রটি বাংলা ভাষায় পড়তে পারি অথবা আপনিও এটি পড়তে পারেন| সম্মতি দেয়ার পূর্বে আপনার যদি কোন প্রশ্ন থাকে তাহলে জিজ্ঞাসা করতে পারেন|

গবেষণার উদ্দেশ্যেঃ

- DAB থেকে BMCH (এবং তদ্বিপরীত) বিদ্যমান রেফারেল পথটি জোরদার করার জন্য সুপারিশ গুলি আপনার কাছে জানতে চাই|
- এই মুহূর্তে, আমরা দেখেছি যে আপনার মতই বরিশাল ড্যাব সেন্টারের কিছু নিবন্ধিত ডায়াবেটিসের রোগী আছেন যাদের বরিশাল মেডিকেল কলেজ ও হাসপাতালের চক্ষু সেবা ইউনিটে পাঠানে হয়েছে কিন্তু তাদের অ্যাপয়েন্টমেন্ট অনুযায়ী তারা সেখানে আসেন নাই|
- পরিশেষে, এই গবেষণাটি একটি বইয়ের অংশ হিসাবে প্রকাশিত হতে পারে অথবা একটি জার্নাল অংশ হিসাবে পক্র াশিত হতে পারে|

গবেষণা পদ্ধতির বর্ণনাঃ

- যদি-আপনি এই গবেষণায় অংশ নিতে সম্মত হন, তবে আপনার ডায়াবেটিস সম্প র্কে সচেতনতা /জ্ঞান এবং ড্যাব সেন্টার থেকে পাওয়া তথ্য এবং দৃষ্টি সমস্যার সম্প র্কে আপনার বোঝার কথা বলতে বলা হবে| এই ইন্টারভিউ সম্পূ র্ণ করতে প্রায় ৪০ মিনিট সময় লাগবে|

এই গবেষণার ঝুঁকিঃ কোন যুক্তিসঙ্গত প্রত্যাশিত ঝুঁকি নেই কেননা এইখানে কোনও মেডিকেল হস্তক্ষেপ থাকবে না|

অধ্যয়নরত থাকার উপকারিতাঃ

- এই সাক্ষাত্কারের সময় আপনার প্রদত্ত তথ্য এবং যে দৃষ্টিভঙ্গিগুলি আপনি দেখিয়েছেন - তা দিয়ে নীতিমালা ও স্টেকহোল্ডারদের চোখের রোগীদের জন্য রেফারেল সিস্টেমকে বিকাশ ও জোরদার করার সুপারিশ করা হবে| এভাবে এই সাক্ষাতকারের কারণে বৃহত্তর সম্প্র্দায় সুবিধা পাবে|

গোপনীয়তাঃ

- এই গবেষণা বেনামী হয়| আমরা আপনার পরিচয় সম্পর্কে কোন তথ্য সংগ্রহ করব না| এই গবেষণার রেকর্ড কঠোরভাবে গোপনীয় রাখা হবে| রিসার্চ রেকর্ড একটি লক করা ফাইলে রাখা হবে|
- আমরা কোন তথ্য অন্তর্ভর্ক্তূ করব না যা আপনাকে সনাক্ত করতে পারে|
- আপনার পরিচয় কোন প্রকাশনার মাধ্যমে প্রকাশ করা হবে না|

পেমেন্টস্ঃ

- এই সাক্ষাত্কারের সময় আপনি যে মূল্যবান সময় প্রদান করবেন তার জন্য আপনাকে কোনও আর্থিক সুবিধা বা অর্থপ্রদান করা হবে না| সময় এবং আপনার প্রদান করা তথ্য অনযুায়ী আপনার অবদান সম্পূর্ণভাবে স্বেচ্ছাসেবী হবে, যা আপনার বৃহত্তর সম্প্র্দায়কে উপকৃত করবে|

প্রত্যাহারের অধিকারঃ

- এই গবেষণায় অংশ নেওয়ার সিদ্ধান্ত সম্পূর্ণরূপে আপনার উপরে| আপনি এই গবেষণার তদন্তকারীদের সাথে আপনার সম্পর্ক্ প্রভাবিত না করে যে কোন সময় গবেষণায় অংশ নিতে অস্বীকার করতে পারেন (আপনার সিদ্ধান্তে আপনার কোন রকম ক্ষতি হবে না) আপনি যে কোন সময় ইন্টারভিউ থেকে সম্পূর্ণরূপে নিজেকে প্রত্যাহার করতে পারেন|

সম্মতিঃ

- নীচে আপনার স্বাক্ষর /আঙ্গুলের ছাপটি ইঙ্গিত দেয় যে আপনি এই গবেষণার জন্য একটি স্বেচ্ছাসেবক অংশীদার হিসেবে কাজ করার সিদ্ধান্ত নিয়েছেন এবং আপনি উপরে বর্ণিত তথ্য পড়েছেন এবং বুঝেছেন|

উত্তরদাতা নাম:

উত্তরদাতার স্বাক্ষর: তারিখ:

তদন্তকারীর নাম:

তদন্তকারীর স্বাক্ষর: তারিখ:

সাক্ষীর নাম:

সাক্ষীর স্বাক্ষর: তারিখ:
