# Supplementary material for: Health education improves referral compliance of persons with probable Diabetic Retinopathy: A randomized controlled trial
Source: PLoS One. 2020 Nov 12;15(11):e0242047. doi: 10.1371/journal.pone.0242047 (PMC7660573; doi:10.1371/journal.pone.0242047)
Supplement: S3 File — (PDF) [file pone.0242047.s005.pdf]

Questionnaire for research participants

Name of Enumerator:

Date of interview: __ __ / __ __ / __ __ (DD/MM/YY)

Questionnaire No: __ __ __ ____ __ __ __

Participant's Name: __ __ __ ____ __ __ __

Address: ____ __ __ __(village) ____ ___ _ ___ _ (sub-district)__ ___ ___ ____(district)__ __ __ __

Mobile number of participant: __ __ __ ____ __ __ __ (match with hospital registry)

Respondent ID: __ __ __ ____ __ __ __ (match with hospital registry)

			Code
	Informed Consent Provided	1. Yes<br>2. No	
**Respondent's Profile: Socio Demographic Characteristics**			
1.	Age		
2.	Sex	1. Female<br>2. Male<br>3. Third gender	
3.	Married status	1. Unmarried<br>2. Married<br>3. Widow/widowed<br>4. Divorced<br>5. Separated	
4.	Occupation	1. Farming – Own/Share<br>2. Agricultural Labour<br>3. Non agricultural Labour<br>4. Fishing<br>5. Rickshaw/van puller)<br>6. Motorized transport driver<br>7. Motorized transport worker<br>8. Self employed in cottage industry<br>9. Garments Worker<br>10. Small/petty business<br>11. Big business<br>12. Housewife<br>13. Student<br>14. Job in the private sector<br>15. Job in the public sector<br>16. Working / Living in abroad<br>17. Self Employed<br>18. Disabled/challenged/old, cannot work<br>19. Unemployed<br>20. Domestic helper<br>21. Tailoring<br>22. Mechanic<br>23. Quack doctor<br>24. Traditional Birth Attendant<br>25. Pension Allowance<br>26. Social Safety-net Benefit<br>27. Others _________	
5.	How far did you study, if at all?	1. No education<br>2. Can sign<br>3. Class 1-5 (primary)<br>4. Class 6-10 (secondary)	

		5. SSC passed / Dakhil<br>6. HSC passed / Alim<br>7. Graduate / Fazil<br>8. Masters and higher / Kamil<br>9. Vocational/Polytechnic<br>10. Others (specify) _____	
6	Can you please provide an estimated range or indication of your monthly income?	1. BDT 0-4,999<br>2. BDT 5,000-9,999<br>3. BDT 10,000-14,999<br>4. BDT 15,000-19999<br>5. BDT 20,000 – 49,999<br>6. BDT 50,000+	
**TRANSPORTATION RELATED DISCUSSION**			
7.	How many types/modes of transportation did you have to utilize to travel from your home to DAB centre?	1. One<br>2. Two<br>3. Three<br>4. Four	
8.	How many types/modes of transportation need to be crossed to travel from your home to Barisal Medical College and Hospital?	1. One<br>2. Two<br>3. Three<br>4. Four	
9. c<br>v	What was the total travel cost required for you and any other accompanying person(s) to travel from your home to DAB center?	BDT ______	
10.	How long did it take to travel from your home to the DAB centre?	___ Hours ___ Minutes	
11. c<br>v	What is the total travel cost required for you and any other accompanying person(s) to travel from your home to Barisal Medical College and Hospital?	BDT ______	
12.	How long does it take to travel from your home to Barisal Medical College and Hospital?	___ Hours ___ Minutes	
**HISTORY OF HEALTH SERVICE UPTAKE RELATED TO DM**			
13.	Which diabetic centre do you visit regularly?	1. Barisal DAB<br>2. Patuakhali DAB<br>3. Jhalokathi DAB<br>4. Others (please specify) ______	1
14.	How long have you been receiving services at this DAB center?	___ Years and ___ Months	

15.	Do you have any vision related problem?	1. Yes<br>2. No<br>3. Do not know	
16.	How would you classify your current vision status?	1. Very Good<br>2. Good<br>3. Neutral<br>4. Bad<br>5. Very Bad	
17.	What are the symptoms of your vision problems?	1. None, I can see clearly<br>2. Few black spots<br>3. Many black spots<br>4. White cloud / cataract<br>5. Hazy vision (may be Glaucoma)<br>6. Near-sightedness<br>7. Far-sightedness<br>8. Others ____	
18.	How long ago did you first have vision problems?	___ Years & ___ Months	
19.	When was the last time you had an eye screening?	1. Within last 6 months<br>2. Within last 12 months<br>3. Within last 24 months<br>4. Within last 36 months<br>5. More than 3 years ago	
**QUALITY OF LIFE (QOL)**			
20.	Do you face physical disability because of vision related problems?	1. Strongly Agree<br>2. Agree<br>3. Neutral<br>4. Disagree<br>5. Strongly Disagree	
21.	Do you need someone's assistance to perform day-to-day activities, because eye related problems?	1. Strongly Agree<br>2. Agree<br>3. Neutral<br>4. Disagree<br>5. Strongly Disagree	
**KNOWLEDGE REGARDING DR**			
22.	Do you know that long-term uncontrolled diabetes might be a cause for your vision problem known as DR?	1. Yes<br>2. No	
23.	Do you know the symptoms of DR?	1. Yes<br>2. No	
24.	What are the symptoms of DR?<br><br>*(Multiple answers acceptable. Put comma between codes)*	1. Few black spots<br>2. Many black spots<br>3. White cloud (maybe cataract)<br>4. Hazy vision (may be Glaucoma)<br>5. Near-sightedness	

		6. Far-sightedness<br>7. Others (specify) _______<br>8. Do not know	
25.	From whom did you come to know that diabetes may cause vision problems?,	1. Government's Community Health Workers<br>2. DAB centre health service providers<br>3. Pharmacist<br>4. Awareness campaign (please specify)<br>5. Private health facility<br>6. Family member<br>7. Neighbour/relative/friend<br>8. Other (specify)	
26.	Do you think onset of DR can be delayed?	1. Yes<br>2. No<br>3. Do not know	
27.	Do you think DR can be prevented?	4. Yes<br>5. No<br>6. Do not know	
28.	Do you know how the onset of DR can be delayed?<br><br>(Multiple answers acceptable. Put comma between codes)	1. Control blood sugar<br>2. Take regular medicine<br>3. Take regular insulin<br>4. Exercise regularly<br>5. Diet control<br>6. Conduct eye screening for DR every 6 months<br>7. Conduct eye screening for DR every 12 months<br>8. Others (specify)________	
29.	Do you think DR can be treated?	1. Yes<br>2. No<br>3. Do not know	
30.	How can DR be treated?<br><br>(Multiple answers acceptable. Put comma between codes)	1. Eye operation<br>2. Laser surgery<br>3. Eye drops<br>4. Oral medicines<br>5. Injection (e.g. Avastin)<br>6. Quack doctors<br>7. Herbal remedy<br>8. Others (specify)	
**SERVICES RECEIVED AT DAB CENTER**			
31.	At the DAB center, did some health personnel carry out your vision screening?	1. Yes<br>2. No<br>3. Do not remember	

32.	For how long did that screening last?	___ Minutes	
33.	For how long did the health personnel counsel you after vision screening?	___ Minutes	
34.	What eye care related information did s/he give you, if any?	(Open-ended questionnaire)<br><br>Try to understand whether service provider informed patient if DR was treatable. Do not use leading question such as "Did a health service provider inform you that DR can be treated".<br><br>1. DR can be treated<br>2. DR cannot be treated<br>3. Did not provide eye care related information<br><br>Note other information provided as well.	
35.	Did s/he give you information about how to control diabetes?	1. Yes<br>2. No<br>3. Cannot remember	
36.	If yes to above, how were you advised to control diabetes?<br><br>*(Multiple answers acceptable. Put comma between codes)*	1. Maintaining diet<br>2. Exercise<br>3. Regular intake of medicine<br>4. Regular use of insulin<br>5. Regularly visit diabetes clinic<br>6. Others (specify) _________	
37.	Did some health personnel refer you to an Eye Doctor?	1. Yes<br>2. No	
38.	(If answer to above question is yes):<br><br>Which eye care facility were you referred to?	Please write the name of referral center	
39.	Were you provided with other information regarding eye care services at referred facility? If yes, which are those?	1. Time to travel to eye facility<br>2. Mode of travel to eye facility<br>3. Cost of services at eye facility<br>4. Eye Consultant's service delivery days<br>5. Eye Consultant's DR Management service delivery timing<br>6. Skills/training of the Eye Consultant	

		7. Information relating to advanced DR screening and treatment procedures<br>8. Ideal interval time before eye screening for the specific patient	
40.	Was the referral system clear?	1. Yes<br>2. No	
41.	If no to above, what was not clear about the referral system?	(Open-ended questionnaire)	
42.	Can you please categorize your experience in dealing with the eye care health personnel at the DAB center?	1. Very good<br>2. Good<br>3. Neutral<br>4. Bad<br>5. Very bad	
43.	How much time did you have to wait to access eye care services, i.e. at the eye OPD?	______ Minutes	
44.	Could you easily understand the language of the eye health care provider?	1. Yes<br>2. No	
**REFERRAL AND FOLLOW-UP**			
45.	Were you provided with a referral slip at the DAB to visit an Eye Consultant?	1. Yes<br>2. No	
46.	Do you still have it preserved?	1. Yes<br>2. No<br><br>(If yes, Interviewer to take a picture of the referral slip)	
47.	Did you receive phone calls as reminders for your appointment with Eye Consultant?	1. Yes<br>2. No	
48.	(If yes to above question)<br><br>How many times?	1. Never<br>2. Once<br>3. Many times	
49.	How many days after referral?	___ days after referral	
50.	Did you visit the same DAB center after you were referred to an Eye Consultant?	1. Yes<br>2. No	

51.	(If yes to above question)<br><br>• Did the health provider ask you whether you visited an Eye Consultant as per past advice?<br>• What did the health provided tell you then?<br>• For how long did this conversation last?	(Open-ended questionnaire)	
**DECISION MAKING (ACCOMPANYING PERSON)**			
52.	Did you attend your referral appointment	1. Yes<br>2. No	
53.	(if yes to above):<br><br>Was it you or an accompanying person who really made the decision to visit/not visit an Eye Consultant?	1. Respondent (self)<br>2. Spouse<br>3. Children<br>4. Other family members<br>5. Friend<br>6. Neighbour<br>7. Others ______	
54.	Why did you/did you not visit the Eye Consultant at the referral facility (i.e. tertiary hospital)?<br><br>*(Please tell me the reasons according to your priority)*	(Open-ended questionnaire; take notes and list top 4 reasons for compliance or non-compliance according to respondent's priority order)<br><br>1. __________________<br>2. __________________<br>3. __________________<br>4. __________________	
**PERCEPTION ABOUT OWN VISION**			
55.	Do you understand the impact of non-compliance on your vision?	1. Yes<br>2. No	
56.	If yes to above question<br><br>What do you think will happen to your vision in case of non-compliance?	(Open-ended Questionnaire)	
57.	Do you think that it is important to visit an Eye Consultant to get your eyes checked?	1. Strongly Agree<br>2. Agree<br>3. Neutral<br>4. Disagree<br>5. Strongly Disagree	
**KNOWLEDGE ABOUT AVAILABLE EYE CARE SERVICES FOR PATIENTS WITH DIABETES**			
58.	Do you know that a skilled Eye Consultant who is trained in DR Management provides services at minimal cost at Barisal Medical	1. Yes<br>2. No	

	College & Hospital?		
59.	Do you know the days and timings of DR screening and treatment at Barisal Medical College and Hospital?	1. Yes<br>2. No	
**Preference About Reminder Schedule**			
60.	Will you be interested to get eye screening at your next visit to the diabetes hospital?	1. Yes<br>2. No<br>3. Neutral	
61.	Would it helpful if you are reminded of your pending dilated eye screening at the tertiary hospital?	4. Yes<br>5. No<br>6. Neutral	
62.	If yes, which mode is more preferable for you?	1. Phone calls<br>2. SMS<br>3. Verbal reminder on next DAB visit<br>4. Through community health workers<br>5. Community Radio<br>6. Others ____	
**Willingness to pay**			
63.	Are you willing to pay a minimal cost for eye screening at the DAB centers?	1. Yes<br>2. No<br>3. Neutral	
64.	If yes, how much are you willing to pay?	1. BDT 0 – 50<br>2. BDT 51 – 100<br>3. BDT 101 – 150<br>4. BDT 151 – 200<br>5. BDT 201 – 400<br>6. BDT 401 +	
**Support / Collaboration**			
65.	What steps do you think health facilities can take to increase eye screening among persons with diabetes?	(Open-ended Questionnaire)	
66.	What support do you expect from government, NGOs or private organizations (such as DAB) to help persons with diabetes overcome the barriers to accessing eye care services?	(Open-ended questionnaire)	

Thank respondent for his/her valuable time and inputs.
