# Supplementary material for: Health education improves referral compliance of persons with probable Diabetic Retinopathy: A randomized controlled trial
Source: PLoS One. 2020 Nov 12;15(11):e0242047. doi: 10.1371/journal.pone.0242047 (PMC7660573; doi:10.1371/journal.pone.0242047)
Supplement: S4 File — (PDF) [file pone.0242047.s006.pdf]

এপেন্ডিক্স-১ (নিবন্ধনভুক্ত ডায়াবেটিক রোগী)

প্রশ্নকারীর নাম :

সাক্ষাৎকারের তারিখ : .... /........ /........ প্রশ্নপত্রের নং :

ঠিকানা : .... (গ্রাম) .... (উপজেলা) .... (জেলা)

উত্তরদাতার নাম : ....উত্তরদাতার মোবাইল নং : .... (হাসপাতালের রেজিস্ট্রি এর সাথে মিলিয়ে)

			কোড
	সম্মতি দিয়েছেন	১. হ্যা ২. না	
RESPONDENT'S PROFILE: SOCIO DEMOGRAPHIC CHARACTERISTICS			
1.	বয়স		-
2.	লিঙ্গ	১. পরুষ<br>২. মহিলা<br>৩. তৃতীয় লিঙ্গ	
3.	বৈবাহিক অবস্থা	১. অবিবাহিত<br>২. বিবাহিত<br>৩. বিধবা/ বিপত্নীক<br>৪. তালাকপ্রাপ্ত<br>৫. বিভক্ত/ ছাড়াছাড়ি	
4.	পেশা	১. কৃষক - নিজস্ব / শেয়ার<br>২. কৃষি শ্রম<br>৩. অ-কৃষি শ্রম<br>৪. মাছধরা<br>৫. রিক্সা / ভ্যান চালক<br>৬. পরিবহন ড্রাইভার<br>৭. পরিবহন কর্মী<br>৮. কুটির শিল্প<br>৯. গার্মেন্টস শ্রমিক<br>১০. ছোট / ক্ষুদ্র ব্যবসা<br>১৬. বিদেশে কাজ / বসবাস<br>১৭. স্বয়ং নিয়োগ<br>১৮. নিষ্ক্রিয় / কাজ করতে পারে না<br>১৯. বেকার<br>২০. ঘরোয়া সাহায্যকারী<br>২১. সেলাই কাজ<br>২২. মেকানিক<br>২৩. ঝাড় ফুঁ (কোয়েক ডাক্তার)	

		১১. বড় ব্যবসা<br>১২. গৃহিনী<br>১৩. ছাত্র<br>১৪. বেসরকারী খাতে কাজ<br>১৫. পাবলিক সেক্টরে কাজ<br>২৪. ঞৎধফরঃরড়হধষ ইরৎঃয অঃঃবহফধহঃ<br>২৫. পেনশন ভাতা<br>২৬. সামাজিক নিরাপত্তা-নেট বেনিফিট<br>২৭. অন্যান্য (উল্লেখ করুন) ________	
5.	আপনি কতদুর পড়াশোনা করেছেন ?	১. শিক্ষা নেই<br>২. সাইন করতে পারেন<br>৩. ক্লাস 1-5 (প্রাথমিক)<br>৪. ক্লাস 6-10 (মাধ্যমিক)<br>৫. এসএসসি পাস / দাখিল<br>৬. এইচএসসি পাস / আলীম<br>৭. স্নাতক (Graduate) / ফাজিল<br>৮. মাস্টারস এবং উচ্চতর (Masters and above) / কামিল<br>৯. পেশাগত / পলিটেকনিক (Vocational/Polytechnic)<br>১০. অন্যান্য (উল্লেখ করুন):________________	
6.	আপনার আনুমানিক মাসিক আয় কত?	১. 0-4,999 টাকা<br>২. 5,000-9,999 টাকা<br>৩. 10,000-14,999 টাকা<br>৪. 15,000-19,999 টাকা<br>৫. 20,000-49,000 টাকা<br>৬. 50,000 + টাকা	
TRANSPORTATION RELATED DISCUSSION			
7.	ডায়াবেটিক হাসপাতালে যেতে আপনার বাড়ি থেকে কয় প্রকার পরিবহন পারি দিতে হয়েছে?<br>**(উদাহরণস্বরূপ: শুধুমাত্র যদি বাস হয় কোড হবে ১; যদি বাস এবং ফেরি হয় কোড হবে ২)**	১. এক<br>২. দুই<br>৩. তিন<br>৪. চার	

8.	বরিশাল মেডিকেল হাসপাতালে যেতে আপনার বাসা থেকে কয় প্রকারের পরিবহন পারি দিতে হয়েছে?	১. এক<br>২. দুই<br>৩. তিন<br>৪. চার	
9.	বাড়ি থেকে ডায়াবেটিক হাসপাতালে যাওয়ার জন্য আপনার এবং আপনার সহকারীকে মোট কত টাকা ব্যয় করতে হয়েছে?	______ টাকা	-
10.	আপনার বাড়ি থেকে ডায়াবেটিক হাসপাতালে যেতে কত সময় লেগেছে?	______ ঘন্টা ______ মিনিট	-
11.	বাড়ি থেকে বরিশাল মেডিকেল হাসপাতালে যাওয়ার জন্য আপনার এবং আপনার সহচারীকে মোট কত টাকা ব্যয় করতে হয়েছে?	______ টাকা	-
12.	আপনার বাড়ি থেকে বরিশাল মেডিকেল হাসপাতালে যেতে কত সময় লেগেছে?	______ ঘন্টা ______ মিনিট	-
HISTORY OF HEALTH SERVICE UPTAKE RELATED TO DM			
13.	আপনি কোন ডায়াবেটিক হাসপাতালে নিয়মিত যান?	১. বরিশাল DAB<br>২. পটুয়াখালী DAB<br>৩. ঝালকাঠি DAB<br>৪. অন্যান্য DAB (উল্লেখ করুন) __________	
14.	আপনি কতদিন যাবত এই ডায়াবেটিক হাসপাতালের সেবা গ্রহণ করছেন?	____ বছর এবং _____মাস	-
15.	আপনার চোখে কোন সমস্যা অনুভব করেন?	১. হ্যাঁ<br>২. না<br>৩. জানি না	
16.	আপনার দৃষ্টিশক্তির অবস্থা আপনি কিভাবে শ্রেণীভুক্ত করবেন?	১. খুব ভাল (Very Good)<br>২. ভাল (Good)<br>৩. নিরপেক্ষ (Neutral)<br>৪. খারাপ (Bad)<br>৫. খুব খারাপ (Very Bad)	

17.	আপনার চোখের সমস্যার লক্ষণগুলো বলুন ?	১. সমস্যা নেই, আমি পরিষ্কার ভাবে দেখতে পারি<br>২. কিছু কালো দাগ<br>৩. অনেক কালো দাগ<br>৪. ছানি / সাদা মেঘ<br>৫. অস্পষ্ট দৃষ্টি (Glaucoma হতে পারে)<br>৬. নিকটদৃষ্টি (- power)<br>৭. দূরদৃষ্টি (+ power)<br>৮. অন্যান্য (উল্লেখ করুন)__________	
18.	আপনার চোখের সমস্যা কতদিন থেকে অনভুব করছেন?	_______বছর এবং _______মাস	-
19.	আপনি শেষবার চোখ পরীক্ষা করেছেন কবে?	১. গত 6 মাসের মধ্যে<br>২. গত 12 মাসের মধ্যে<br>৩. গত 24 মাসের মধ্যে<br>৪. গত 36 মাসের মধ্যে<br>৫. তিন বছর পূর্বে	
**QUALITY OF LIFE (QOL)**			
20.	চোখের সমস্যার কারণে কি শারীরিক সমস্যা হয়?	১. **দৃঢ়ভাবে সম্মত** (Strongly agree)<br>২. **সম্মত** (Agree)<br>৩. **নিরপেক্ষ** (Neutral)<br>৪. **অসম্মত** (Disagree)<br>৫. **দৃঢ়ভাবে অসম্মতি** (Srongly disagree)	
21.	আপনার কি চোখের সমস্যার কারনে প্রতিদিনের কার্যক্রমে কারো সহযোগিতা প্রয়োজন হয়?	১. **দৃঢ়ভাবে সম্মত** (Strongly agree)<br>২. **সম্মত** (Agree)<br>৩. **নিরপেক্ষ** (Neutral)<br>৪. **অসম্মত** (Disagree)<br>৫. **দৃঢ়ভাবে অসম্মতি** (Srongly disagree)	
**KNOWLEDGE REGARDING DR**			
22.	আপনি কি জানেন যে দীর্ঘদিন ডায়াবেটিস অনিয়ন্ত্রিত থাকলে, একটি চক্ষু সমস্যা দেখা দিতে পারে, যার নাম ডায়াবেটিক রেটিনোপ্যাথি?	১. হ্যাঁ<br>২. না	
23.	আপনি কি ডায়বেটিক রেটিনোপ্যাথির লক্ষণগুলো সম্পর্কে জানেন?	১. হ্যাঁ<br>২. না<br>৩. জানি না	

24.	ডায়াবেটিসের রেটিনোপ্যাথি লক্ষণগুলো বলুন...<br><br>(একাধিক উত্তর গ্রহণযোগ্য। কোডের মাঝে কমা রাখুন)	১. কিছু কালো দাগ<br>২. অনেক কালো দাগ<br>৩. ছানি / সাদা মেঘ<br>৪. অস্পষ্ট দৃষ্টি (Glaucoma হতে পারে)<br>৫. নিকটদৃষ্টি (- power)<br>৬. দূরদৃষ্টি (+ শক্তি)<br>৭. অন্যান্য __________<br>৮. জানি না	
25.	আপনি প্রথম কার মাধ্যমে শুনেছেন যে ডায়াবেটিসের কারণে চোখের সমস্যা হতে পারে| <br>(একাধিক উত্তর গ্রহণযোগ্য। কোডের মাঝে কমা রাখুন)	১. সরকারের কমিউনিটি স্বাস্থ্যকর্মী<br>২. DAB সেন্টার স্বাস্থ্য সেবা প্রদানকারীরা<br>৩. ফার্মাসিস্ট<br>৪. সচেতনতা প্রচারণা (নির্দিষ্ট করুন): __________________<br>৫. ব্যক্তিগত স্বাস্থ্য সুবিধা<br>৬. পরিবারের সদস্য<br>৭. প্রতিবেশী / বন্ধু<br>৮. অন্যান্য (নির্দিষ্ট করুন) ________________	
26.	ডায়াবেটিক রেটিনেপ্যথি হওয়াটা **বিলম্বিত (দেরি)** করা যায় কি না -<br>এ ব্যপারে আপনি কি মনে করেন?	১. হ্যাঁ - করা যেতে পারে<br>২. **না - করা যাবে না**<br>৩. **জানি না**	
27.	ডায়াবেটিক রেটিনোপ্যথির **প্রতিরোধ** করা যায় কি না - এ ব্যপারে আপনার কি ধারনা?	১. হ্যাঁ - করা যেতে পারে<br>২. **না - করা যাবে না**<br>৩. **জানি না**	
28.	ডায়াবেটিক রেটিনোপ্যথির **কিভাবে প্রতিরোধ করা যায়**, আপনার কি তা জানা আছে?<br><br>(একাধিক উত্তর গ্রহণযোগ্য। কোডের মাঝে কমা রাখুন)	১. রক্তে Sugar নিয়ন্ত্রণ করতে হবে<br>২. নিয়মিত ঔষধ<br>৩. নিয়মিত ইনসুলিন<br>৪. নিয়মিত ব্যায়াম<br>৫. খাদ্য নিয়ন্ত্রণ<br>৬. প্রতি 6 মাসে ডিআরের জন্য চোখের স্ক্রিনিং করতে হবে<br>৭. প্রতি 12 মাসে ডিআরের জন্য চোখের স্ক্রীনিং করতে হবে<br>৮. অন্যান্য (নির্দিষ্ট করুন) __________________	

29.	ডায়াবেটিক রেটিনেপ্যতিকর **চিকিৎসা** করা যায় কিনা এ ব্যপারে আপনার কি ধারনা?	১. হ্যাঁ - করা যেতে পারে<br>২. না - করা যাবে না<br>৩. জানি না	
30.	ডায়াবেটিক রেটিনেপ্যথির **চিকিৎসা কিভাবে করা যেতে পারে**?<br>(একাধিক উত্তর গ্রহণযোগ্য। কোডের মাঝে কমা রাখুন)	১. চোখের অপারেশন<br>২. লেসার সার্জারি<br>৩. চোখের ড্রপ<br>৪. মৌখিক ওষুধ<br>৫. ইঞ্জেকশন (উদাঃ Avastin)<br>৬. ঝাড় ফুঁ (কোয়েক ডাক্তার)<br>৭. হার্বাল প্রতিকার<br>৮. অন্যদের (নির্দিষ্ট করুন)__________________	
SERVICES RECEIVED AT DAB CENTER			
31.	ডায়াবেটিস হাসপাতালে স্বাস্থ্যকর্মী কি আপনার চোখ পরীক্ষা করেছেন?	১. হ্যাঁ<br>২. না	
32.	যদি করে থাকে, কতক্ষণ ধরে আপনার চোখ পরীক্ষা করা হল?	__________মিনিট	-
33.	তারপর কতক্ষণ যাবত আপনাকে স্বাস্থ্যকর্মী পরামর্শ দিলেন?	__________মিনিট	-
34.	• DR চিকিৎসা করা যেতে পারে/ পারে না - আপনাকে স্বাস্থ্যকর্মী কোন তথ্য দিয়েছেন?<br>• আপনাকে স্বাস্থ্যকর্মী চোখের ব্যাপারে আর কোন তথ্য দিয়েছেন?	(সবিস্তার প্রশ্নাবলী) সেবা প্রদানকারী রোগীকে DR ব্যাপারে কোন কোন তথ্য দিয়েছেন তা বুঝতে চেষ্টা করুন। Leading প্রশ্ন ব্যবহার করবেন না।<br>১. DR চিকিৎসা করা যেতে পারে<br>২. DR চিকিৎসা করা যাবে না<br>৩. চোখের যত্ন সম্পর্কিত তথ্য প্রদান করা হয়নি<br>অন্যান্য তথ্য নোট: ____<br>____	
35.	আপনাকে কি স্বাস্থ্যকর্মী ডায়াবেটিস নিয়ন্ত্রণের ব্যপারে কোন তথ্য দিয়েছেন?	১. হ্যাঁ<br>২. না<br>৩. মনে করতে পারছি না	

36.	যদি দিয়ে থাকেন, কিভাবে ডায়াবেটিস নিয়ন্ত্রণ করা যায় বলে আপনাকে জানিয়েছেন?<br><br>(একাধিক উত্তর গ্রহণযোগ্য উত্তর; কোডের মাঝে কমা রাখুন)	১. খাদ্য নিয়ন্ত্রণ<br>২. ব্যায়াম<br>৩. নিয়মিত ঔষধ খাওয়া<br>৪. ইনসুলিন নিয়মিত ব্যবহার<br>৫. নিয়মিত ডায়াবেটিস ক্লিনিকে যেতে হবে<br>৬. অন্যান্য (নির্দিষ্ট করুন)________________	
37.	আপনাকে কি স্বাস্থ্যকর্মী চক্ষু ডাক্তারের কাছে যেতে বলেছেন?	১. হ্যা ২. না	
38.	যদি বলে থাকেন, কোন চক্ষু হাসপাতালে যেতে বলেছেন?	রেফারেল সেন্টার নাম লিখুন: _______________	
39.	রেফারকৃত হাসপাতালের চক্ষু সেবা সম্পর্কে আপনাকে অন্যান্য তথ্য কি দেয়া হয়েছিল?<br><br>(একাধিক উত্তর গ্রহণযোগ্য উত্তর; কোডের মাঝে কমা রাখুন)	১. DAB থেকে সেখানে যেতে কত সময় প্রয়োজন<br>২. কিভাবে হাসপাতালে পৌঁছাতে হবে<br>৩. চোখের সেবার খরচ<br>৪. চোখের সেবার দিন<br>৫. উজ সার্ভিস ডেলিভারির সময়<br>৬. আই কনসালটেন্টের দক্ষতা / প্রশিক্ষণ<br>৭. উন্নত DR স্ক্রীনিং এবং চিকিত্সা পদ্ধতি সম্পর্কিত তথ্য<br>৮. সেখানে কত শীঘ্র যেতে হবে	
40.	রেফার করার পদ্ধতি কি আপনার কাছে পরিষ্কার হয়েছিল?	১. হ্যাঁ<br>২. না	
41.	যদি না হয়ে থাকে কোন বিষয়টি পরিস্কার হয়নি?	(সবিস্তার প্রশ্নাবলী):	-
42.	ডায়াবেটিস হাসপাতালের চক্ষু সেবা প্রদানকারীর সাথে আপনার অভিজ্ঞতা কিভাবে শ্রেণীভূক্ত করবেন?	১. খুব ভাল (Ver Good)<br>২. ভাল (Good)<br>৩. নিরপেক্ষ (Neutral)<br>৪. খারাপ (Bad)<br>৫. খুব খারাপ (Very Bad)	
43.	ডায়াবেটিস হাসপাতালে চক্ষু সেবা নেয়ার আগে কতক্ষণ আপনাকে অপেক্ষা করতে হয়েছিল?	________ মিনিট	-
44.	চক্ষু সেবা প্রদানকারীর ভাষা ও বোঝানোর ক্ষমতা কি আপনার কাছে পরিস্কার ছিল?	১. হ্যাঁ<br>২. না	

REFERRAL AND FOLLOW-UP			
45.	চক্ষু ডাক্তারের সাথে দেখা করার জন্য আপনাকে ডায়াবেটিক হাসপাতাল থেকে কোন স্লিপ/কার্ড দিয়েছিল?	১. হ্যাঁ<br>২. না	
46.	আপনার কাছে কি এখনো সেই স্লিপ/কার্ড আছে?	১. হ্যাঁ (এই ক্ষেত্রে, রেফারেল স্লিপ-এর একটি ছবি গ্রহণ করুন<br>২. না	
47.	চক্ষু ডাক্তারের সাথে দেখা করার জন্য আপনাকে কেউ ফোন করেছিল?	১. হ্যাঁ<br>২. না	
48.	কতবার ফোন করেছিল?	১. কখনও না<br>২. একবার<br>৩. অনেক বার	
49.	রেফারেল-এর কত দিন পরে ফোন করেছিল?	_______ দিন পরে	
50.	চক্ষু ডাক্তারের কাছে রেফার করার পর আপনি কি ঐ একই ডায়াবেটিক হাসপাতালে (DAB) গিয়েছিলেন?	১. হ্যাঁ<br>২. না	
51.	(যদি উপরে প্রশ্নের উত্তর হ্যাঁ হয়)<br>• ডায়াবেটিস হাসপাতাল পরের বার যাওয়া পরে স্বাস্থ্যকর্মী আপনার কাছে রেফারকৃত চক্ষু ডাক্তারের কাছে যাওয়ার ব্যাপারে কিছু জানতে চেয়েছিল কিনা?<br>• তখন -স্বাস্থ্যকর্মী আপনাকে কি বলেছিল?<br>• কত সময় নিয়ে বলেছিল	(সবিস্তার প্রশ্নাবলী)<br>• ________ (হ্যাঁ/না)<br>• ________________________________<br>________________________________<br>________________________________<br>________________________________<br>• ________ মিনিট	-
DECISION MAKING (ACCOMPANYING PERSON)			
52.	রেফারেল অনুযায়ী আপনি কি বরিশাল মেডিকেলে চক্ষু ডাঃ সাথে দেখা করেছিলেন?	১. হ্যাঁ<br>২. না	
53.	**(যদি #৫২ প্রশ্নের উত্তর হ্যাঁ হয়)**<br>বরিশাল মেডিকেলে চক্ষু ডাঃ সাথে কি আপনি নিজে থেকে দেখা করেছেন, নাকি পরিবারের সদস্য/বন্ধু আপনাকে সেখানে নিয়ে গিয়েছিলেন?	১. নিজের থেকে<br>২. স্বামী বা স্ত্রী<br>৩. সন্তান<br>৪. পরিবারের অন্যান্য সদস্য<br>৫. বন্ধু<br>৬. প্রতিবেশী<br>৭. অন্যান্য ব্যক্তি	

54.	কোন কারণে আপনি বরিশাল মেডিকেলে চোখের ডাক্তারের কাছে যান/ যাননি?<br>[উদাহরণ: যারা SBMCH পরিদর্শন করেছিল তাদের জন্য- **আমার পরিবারের সদস্যরা সহায়ক ছিল, আমার চোখে সমস্যা ছিল;** যারা SBMCH যাননি তাদের জন্য- **সময় পাওয়া যায় না, চোখের যত্নের গুরুত্ব বুঝতে পারিনি**]	(বিস্তারিত লিখুন; **অগ্রাধিকার** অনুসারে কারণগুলির তালিকা গ্রহণ করুন):<br>১. ____________________<br>২. ____________________<br>৩. ____________________<br>৪. ____________________	
PERCEPTION ABOUT OWN VISION			
55.	বরিশাল মেডিকেলে **চক্ষু** ডাঃ সাথে দেখা না করলে আপনার চোখের যে ক্ষতি হতে পারে / পারত সেটা কি আপনি বুঝতে পারেন?	১. হ্যাঁ<br>২. না	
56.	বরিশাল মেডিকেলে **চক্ষু** ডাঃ এর কাছে না গেলে, আপনার চোখের কেমন সমস্যা হত/হবে বলে মনে হয়?	(বিস্তারিত লিখুন)	
57.	আপনার কি মনে হয় যে বরিশাল মেডিকেলে চক্ষু ডাঃ সাথে দেখা করে চোখ পরিক্ষা করা জরুরি?	১. খুব জরুরি<br>২. জরুরি<br>৩. **নিরপেক্ষ**<br>৪. জরুরি না<br>৫. মোটেই জরুরি না	
KNOWLEDGE ABOUT AVAILABLE EYE CARE SERVICES FOR PATIENTS WITH DIABETES			
58.	আপনি কি জানেন বরিশাল মিডিকেলে লে একজন প্রশিক্ষণ প্রাপ্ত চক্ষু ডাঃ বসেন?	১. হ্যাঁ<br>২. না	
59.	বরিশাল মিডিকেলে চক্ষু ডাঃ **কোন দিন কোন সময়ে** বসে সেটা কি আপনি জানেন?	১. হ্যাঁ<br>২. **না**	
PREFERENCE ABOUT REMINDER SCHEDULE			
60.	আপনি পরের বার যখন ডায়বেটিস হাসঃ যাবেন তখন আবার চোখ পরিক্ষা করাতে ইচ্ছুক কিনা?	১. হ্যাঁ<br>২. **না**<br>৩. **নিরপেক্ষ** (Neutral)	
61.	চক্ষু পরিক্ষার জন্য আপনাকে ফোন করে মনে করিয়ে দিলে আপনার কি সাহায্য হবে?	১. হ্যাঁ<br>২. **না**<br>৩. **নিরপেক্ষ** (Neutral)	

62.	আপনাকে কিভাবে মনে করিয়ে দিলে আপনার জন্য সহজ হয়?	১. ফোন কল<br>২. এসএমএস<br>৩. আপনার পরবর্তী DAB পরিদর্শন যখন অনুস্মারক<br>৪. কমিউনিটি স্বাস্থ্য কর্মীদের মাধ্যমে<br>৫. কমিউনিটি রেডিও<br>৬. অন্যান্য (দয়া করে নির্দিষ্ট করুন)_______________________________	
**WILLINGNESS TO PAY**			
63.	ডায়াঃ হাসঃ কিছু টাকা দিয়ে চক্ষু পরিক্ষা করাতে আপনি ইচ্ছুক কিনা?	১. হ্যাঁ<br>২. না<br>৩. নিরপেক্ষ (Neutral)	
64.	কত টাকা পর্যন্ত আপনি দিতে ইচ্ছুক?	১. 0 – 50 টাকা<br>২. 51 – 100 টাকা<br>৩. 101 – 150 টাকা<br>৪. 151 – 200 টাকা<br>৫. 201 – 400 টাকা<br>৬. 401 + টাকা	
**SUPPORT / COLLABORATION**			
65.	হাসপাতলগুলি কি করলে ডায়াবেটিক রোগীরা চোখ পরিক্ষা করতে আসবে বলে আপনার মনে হয়?	(বিস্তারিত লিখুন)<br>--<br>--<br>--	
66.	ডায়াঃ রোগীদের চোখের সেবার বাধা অতিক্রম করতে সরকার এনজিও বা বেসরকারী প্রতিষ্ঠানের কাছ থেকে কিকি সাহায্য আপনি আশা করেন?	(বিস্তারিত লিখুন)<br>--<br>--<br>--	

প্রশ্নের উত্তর প্রদান এবং মূল্যবান সময় এর জন্য উত্তরদাতা কে ধন্যবাদ!
