# Supplementary material for: Health education improves referral compliance of persons with probable Diabetic Retinopathy: A randomized controlled trial
Source: PLoS One. 2020 Nov 12;15(11):e0242047. doi: 10.1371/journal.pone.0242047 (PMC7660573; doi:10.1371/journal.pone.0242047)
Supplement: S5 File — (DOCX) [file pone.0242047.s007.docx]

| Timeline | Contents | Tools and modalities | Duration |
| --- | --- | --- | --- |
| Day 1 | **About** **Diabetes**:   - What is diabetes? - What are the symptoms of diabetes? - Who are at risk of diabetes? - How can diabetes be controlled? - Control diabetes by changing food intake, lifestyle and exercise, and taking prescribed medicine regularly   **About Diabetic Retinopathy:**   - What is Diabetic Retinopathy (DR)? - What is the relation between uncontrolled diabetes and eyesight? - Who are at risk of developing DR? - How can someone prevent and/or delay the onset of DR? - Importance of screening of retina for all persons with diabetes - Build on reputation of service facility (mention that highly skilled and trained Ophthalmologist provides services at the referred facility at Barisal Medical College and Hospital   **Clarify Referral System:**   - Provide information on scheduled days and times of DR Management services at referred facility, along with specific location and address of DR Zone at the referred facility, and details of Ophthalmologist (reiterate that referral card will have all this information and should be preserved with care) - Provide information on cost of services (cost of screening retina using a High Resolution Colour Fundus camera at the referred facility is very minimal, whereas it will take thousands of taka to avail this service if patient decides to travel to Dhaka along with an accompanying person (considering overnight travel, food, accommodation and other relevant costs) - Take a verbal commitment from patient about visiting referred facility | Multicomponent interactive face-to-face awareness raising session  Language of communication: Bengali (using local dialect of Barishal highly encouraged)  Female Community Health Worker to lead session where participant is female  Tools used:   1. Colorful pictorial demonstrative flipchart 2. Colorful pictorial brochure 3. Waterproof Referral information card | 30 – 40 minutes |
| Day 7, 30 and 90 | - Self-introduction by caller - Ask whether patient complied with referral guidance (Dilated fundus eye examination undertaken? If yes, note name of hospital) - Summarize the face-to-face discussion from one month earlier - Clarify the scheduled days and times of DR Management services delivery by trained Ophthalmologist at referred facility - Provide information about cost of services - Take a verbal commitment from patient about visiting referred facility - If patient still does not want to attend, ask reason. In this case, try and motivate by repeating the following points: - importance of good vision   - patient falls into risk category (identified as a probable DR patient at diabetic hospital i.e. Barishal Diabetes hospital)   - possibility of patient developing DR although no symptom maybe present at present   - importance of regular screening of retina using High Resolution Colour Fundus camera which is available in the referred facility   - highly skilled and trained Ophthalmologist provides services at the referred facility (i.e. Barisal Medical College and Hospital)   - remind them that laser surgery can stop further deterioration of vision, but cannot treat it completely   - cost of screening retina using a High Resolution Colour Fundus camera at the referral facility is very minimal, whereas it will take thousands of taka to avail this service if patient decides to travel to Dhaka along with an accompanying person (considering overnight travel, food, accommodation and other relevant costs)   - Lastly, try and take verbal commitment again | Telephone call  Language of communication: Bengali (using local dialect of Barishal highly encouraged) | About 15 minutes per phone call |
