# Supplementary material for: Health education improves referral compliance of persons with probable Diabetic Retinopathy: A randomized controlled trial
Source: PLoS One. 2020 Nov 12;15(11):e0242047. doi: 10.1371/journal.pone.0242047 (PMC7660573; doi:10.1371/journal.pone.0242047)
Supplement: S7 File — (PDF) [file pone.0242047.s009.pdf]

ClinicalTrials.gov Protocol Registration and Results System (PRS) Receipt
Release Date: September 6, 2018

ClinicalTrials.gov ID: NCT03658980

Study Identification

Unique Protocol ID: HiroshimaUniversity

Brief Title: Integrating DR Into Mainstream Health System in Bangladesh

Official Title: Integrating Diabetic Retinopathy Into Mainstream Health System in Bangladesh

Secondary IDs:

Study Status

Record Verification: September 2018

Overall Status: Not yet recruiting

Study Start: October 1, 2018 [Anticipated]

Primary Completion: March 31, 2019 [Anticipated]

Study Completion: March 31, 2019 [Anticipated]

Sponsor/Collaborators

Sponsor: Hiroshima University

Responsible Party: Principal Investigator
Investigator: Zara Khair [zkhair]
Official Title: Principal Investigator
Affiliation: Hiroshima University

Collaborators:

Oversight

U.S. FDA-regulated Drug: No

U.S. FDA-regulated Device: No

U.S. FDA IND/IDE: No

Human Subjects Review: Board Status: Pending
Board Name: Bangladesh Medical Research Council
Board Affiliation: Bangladesh Medical Research Council
Phone: 88028819311
Email: info@bmrcbd.org
Address:

BMRC Bhaban, Mohakhali, Dhaka 1212, Bangladesh

Data Monitoring:

Study Description

Brief Summary: Diabetes mellitus is a leading cause of death and disability. Diabetic Retinopathy (DR) is an eye disease that affects the eyesight of about 21.3% persons with diabetes in Bangladesh in a way that is irreversible because of the nature of the disease. According to International Diabetes Federation Diabetes Atlas (5th Edition), in 2011 there were approximately 8.4 million people in the age bracket 20-79 suffering from diabetes in Bangladesh, and this number is projected to double to 16.8 million by 2030. Based on these statistics, and also according to a nationally published report, the number of persons with DR is estimated to be about 1.8 million in Bangladesh.

Taking measures to delay vision loss in persons with diabetes is more cost-effective than its treatment. Timely and effective referral of DR cases to an Ophthalmologist is very important in the prevention of this disease. The objective of this study is to identify reasons of DR referral procedure compliance and non-compliance among registered persons with diabetes in a diabetic clinic and recommend effective strategies to enhance the referral system.

The first phase of study is cross-sectional. Second phase of study is a Randomized Controlled Trial (intervention group to receive home-based health education and control group to receive standard care). It is expected that the number of registered persons with diabetes of Barishal DAB hospital who were referred to an Eye Consultant at a tertiary hospital from September 2017 - August 2018 will be N=300. All 300 participants will be eligible for inclusion in the first phase of the study. This group (est. N=300) will be categorized into compliant (est. N=120) and non-compliant participants (est. N=180). Both groups will be interviewed to understand motivation factors for compliance and de-motivation factors for non-compliance.

In the second phase of the study, the non-compliant group will be categorized further into intervention and control groups following Randomization. The intervention group will receive relevant health education messages on Diabetic Retinopathy and information about the days and times when eye care services are provided at the tertiary hospital (i.e. service availability information). They will be provided with telephonic reminders at Days 7, 30 and 90 after the health education. Then, after a gap of one month from the last telephonic reminder, both control and intervention groups will be interviewed again. In this way this study will conclude whether the health education intervention is an effective way of improving compliance rate of referred DR persons.

All questionnaires will be pre-tested in the study location. All participants will have to sign a detailed Bengali informed consent form. SPSS software will be used for data entry and analysis. Multiple Logistic Regression, along with other tests, will be used to identify variables that significantly influence successful referrals.

Detailed Description: Diabetes is a non-communicable chronic disease associated with abnormally high levels of the glucose in the blood. One of the many complications of diabetes is Diabetic Retinopathy (DR), which results in vision problems in persons suffering from diabetes for many years.

DR is the fifth leading cause of global blindness, affecting an estimated 1.8 billion people and responsible for 4.8% of all causes of blindness. According to International Diabetes Federation (IDF) Atlas, Bangladesh is a least developed country (LDC) with a disproportionately high proportion of people having diabetes. Among all people living with diabetes in the 48 LDCs, about 40% live in Bangladesh, which makes it one of the top 10 countries suffering from diabetes. In Bangladesh, approximately 6.1% of the population aged 20-79 years has diabetes, with approximately 46% of these undiagnosed. According to International Diabetes Federation Diabetes Atlas (5th Edition), in 2011 there

were approximately 8.4 million people in the age bracket 20-79 suffering from diabetes in Bangladesh, and this number is projected to double to 16.8 million by 2030. Diabetic Retinopathy (DR) and Diabetic Macular Edema (DME) are common complications of diabetes that can result in vision loss - the estimated number of people with DR in Bangladesh is about 1.8 million (21.3% of the diabetic persons).

From a patient perspective there is little or no integration between the eye health sector and the diabetes sector. In Bangladesh, care for diabetes-related eye disease is available in very few tertiary level health facilities. There is currently no eye health care available for people with diabetes or those suffering from DR, at the community clinics, primary health care centers or secondary health care centers (The Fred Hollows Foundation, 2015).

An effective referral system is a 'critical component of quality clinical care'. When utilized optimally, it can solve more public health problems in less time by directing cases through an appropriate channel.

DR Screening is very important, because early stages of the disease lack symptoms - when symptoms occur the disease already requires treatment. DR Screening centers may have to refer persons in a timely and effective manner to a DR treatment center. Early detection and timely referral are critical for timely interventions to prevent further deterioration of vision. In the case of DR, an effective referral system is crucial for the success of the disease management. This is because knowledge, awareness and perceived benefit of receiving DR Management services among persons with diabetes is very low, the world over, and more so in Bangladesh. Therefore, in order to make sure that DR Management is effectively integrated into mainstream public health in Bangladesh, it is vital to address one of the most challenging components of DR management, which is, strengthening referral system for persons having DR. If a person with diabetes is not made aware about possible consequences of negligence of eye problem, is not properly counseled to undertake DR treatment and is not made aware of the existing DR referral pathway, the person will in all probability forego treatment, since the perceived benefit of DR treatment is low among persons who lack symptoms.

The Research Question is ‘How can we increase the number of successful referrals of Diabetic Retinopathy persons from a diabetes clinic (DAB center) to an Eye Consultant located at a tertiary hospital?’ The general objective is to recommend health education messages regarding DR and intervention strategies to benefit people who are at risk of developing DR or has already developed DR.

The specific objective is to investigate the effect of a health education intervention on persons with probable Diabetic Retinopathy who did not attend eye screening appointment with an Eye Consultant following referral from a diabetes clinic.

The primary outcome is ‘Increase in Successful Referrals’ and the secondary outcome is ‘Increase in knowledge of Diabetic Retinopathy’.

RATIONALE:

A diabetic clinic should ideally be a 'one-stop-care center' that offers holistic care for people with diabetes. DR persons may be diagnosed or undiagnosed persons with diabetes. Before a person is screened or treated for DR, ideally that person must first be diagnosed with diabetes, and brought under comprehensive diabetes care management. Comprehensive diabetes care management includes management of diseases of organs including kidneys, heart, tooth, brain and eyes. Yet many undiagnosed persons with diabetes who suffer from DR may also walk into eye clinics, requiring urgent attention. In such cases, it is best to manage DR first and then refer the person to a

diabetes clinic, so that person can be brought under comprehensive diabetes management. Controlling and managing diabetes will ensure that the DR condition of person does not deteriorate. Management of diabetes ensures that DR is controlled or prevented. It is a well-established fact that prevention of DR is more cost-effective than its treatment. In order to delay the onset of DR, it is important that referred DR persons from Barisal DAB Center promptly uptakes services at the referred facility, i.e. the tertiary hospital. Referred persons should ideally avail advanced DR management and treatment options without further loss of time.

In the study location (i.e. Barisal district), there are approximately 21000 persons suffering from varying stages of DR.

Sher-e-Bangla Medical College and Hospital (SBMC&H) has been providing eye care services for a long time to the community, and has recently started to provide eye care services to persons suffering from Diabetic Retinopathy. Their services include eye screening and treatment for DR persons. The Eye Consultant in this hospital has received reputed international training. Most of the people who came to seek DR management services at SBMC&H in 2017 and 2018 are persons with diabetes, registered with the Barishal DAB center, who have been referred from Barisal DAB center to SBMC&H (The Fred Hollows Foundation Bangladesh, 2018).

Diabetes clinics are one-stop-care-center for most registered persons with diabetes. Barisal DAB center is visited by thousands of persons with diabetes each year. If a person with diabetes is referred to an Eye Consultant of a tertiary hospital for advanced DR screening and management, most of the referred persons to not avail services in the tertiary hospital, due to lack of awareness, motivation and other barriers. These 'non-compliant' persons maybe assumed to 'drop out' of the health system (The Fred Hollows Foundation Bangladesh, 2018).

Barisal DAB center provides diabetes management services to registered persons with diabetes, and has in recent year started to provide eye care services to persons suffering from DR (The Fred Hollows Foundation Bangladesh, 2017). Its services include screening for probable DR cases. The medical personnel in this hospital has received training to screen for probable DR cases, and then refer these probable cases to an Eye Consultant for advance screening and management (preferably at SBMC&H since it provides DR treatment and is located nearby). From the records from the year 2017 and 2018, it has been found that most referred persons of Barishal DAB center do not avail their appointment at the referred facility (non- compliant persons). These non-compliant persons are probable priority DR persons who need immediate medical attention (this risk group also has diabetes for more than 5 years, and/or a family history of diabetes, and/or a higher range of Body Mass Index along with worsening eye conditions) (The Fred Hollows Foundation Bangladesh, 2018).

Chances are these same registered persons with diabetes (who have been referred from Barisal DAB center to an Eye doctor) will visit Barisal DAB center in future again for continued management of diabetes and other complication management. These high-risk persons can be followed-up with, regarding their eye screening appointment at SBMC&H (The Fred Hollows Foundation Bangladesh, 2018). This is because timely referral is critical for DR persons, in order to delay further deterioration of their vision, and DR persons need to complete the referral cycle in a prompt manner. That is, the number of successful referrals (or completed referrals) is an important indicator.

From a patient perspective, the referral pathway should be clear and specific. Patients require specific information not only about the disease management, but also about the referred facility, such as days and times of services, how to travel to the referred facility, and so on. Through the health education

component in this research, non-compliant persons will be provided with this type of information, which will help them decide when and how to access tertiary level care. In this way, the non-compliance rate may be minimized.

This study will highlight health education messages and possible health education delivery strategies, targeting DR persons.

The first phase of study is cross-sectional. The second phase of study is a Randomized Controlled Trial (intervention group to receive home-based health education and control group to receive standard care).

- Variables used in study: Education, Occupation, Income, Age, Sex, District of residence, DR Stage, time taken to travel to health facility, knowledge of DR, source of information on DR, accompanying person, decision maker, information received at diabetes clinic, counseling received at diabetes clinic, follow up/reminder calls about appointment with Eye Consultant, clarity about referral procedure (place/time/location/how to get there), satisfaction with health facilities, understanding of non-compliance on vision.
- Study Population: Registered persons with diabetes, who are/will be detected with probable DR and referred for specialty DR services at Sher-e-Bangla Medical College and Hospital (both groups will be studied - those who uptake services and those who do not, i.e. compliant and non-compliant persons)
- Inclusion criteria: All diabetic persons registered at Barisal DAB who are referred to SBMC&H for DR management (screening/treatment), from September 2017 - August 2018 (i.e. during these 12 months) are eligible for inclusion in this study. Also, participants have to be atleast 18 years of age and will provide informed consent to be included in the research study. Only those persons will be included who did not undergo a Dilated Fundus Examination (DFE) in last 12 months.
- Exclusion criteria: Persons with diabetes who are registered at Barisal DAB but are not referred to SBMC&H for DR management (screening/treatment), from September 2017 - August 2018 (i.e. during these 12 months) are excluded from this study. Persons below 18 years of age, and those who do not provide informed consent to be included in the research study, are excluded from the study. Persons who have undergone a Dilated Fundus Examination (DFE) in last 12 months will also be excluded from this study.
- Primary Outcome: Increase in Referral Rate (among non-compliant persons, i.e. those who do not avail appointment with an Eye Consultant at a tertiary hospital after being detected with probable DR at a diabetic hospital)
- Secondary Outcome: Increase in knowledge of Diabetic Retinopathy and service availability
- Statistical basis of the sample size: All diabetic persons registered at Barisal DAB who were referred to Barisal Sher-e-Bangla Medical College and Hospital after screening for probable DR (at the DAB clinic) from September 2017 - August 2018 are eligible for inclusion in this study. The total expected number of referred persons referred from September 2017 - August 2018 is expected to be about N=¬300 based on trend analysis (of monthly data from September 2017 - April 2018). All persons will be interviewed (both compliant and non-compliant persons). ¬ This number (N=300) was calculated based on the following parameters: Confidence Interval = 95%; population of probable DR persons in Barisal district = 20,000; Power = 0.8; Standard Deviation = 10%
- Methods of Data Collection: After completion of informed consent forms, face-to-face In-depth Interviews (IDI) with all participants will be conducted.
- Pre-testing: Questionnaires will be pre-tested in the study location

- Data Interpretation: Responses to questions will be coded and entered into SPSS software, which will then be used to analyze and interpret data.
- Statistical Analysis: Frequency distributions will be used to analyze the demographic characteristics of the study population. Multiple Logistic Regression method will be used to identify variables that significantly influence successful and unsuccessful referrals among compliant and non-compliant persons. For the RCT study among non-compliant persons to find impact of health education intervention, the primary outcome (successful referral rate - defined as attendance at referred facility) and secondary outcome (increase in knowledge of Diabetic Retinopathy and service availability) will be compared between both arms. For continuous measures the t-test will be used and for categorical outcomes, chi-square test will be use, when comparing the two arms (i.e. intervention and control groups).

There are no reasonable foreseeable risks in this study. Participants will be made aware about the confidentiality issues, study procedures, modalities, and their rights to refuse to answer/withdraw at any point without any monetary/non-monetary loss. This study is anonymous. Investigator will not be collecting or retaining any information about participant identity. The records of this study will be kept strictly confidential. Publications will not contain any information that may make it possible to identify participants. Participants will not receive any monetary benefits for participating in this study. They will be informed about this before the start of the survey. They will be required to sign a detailed consent form that they can read in Bengali and have a witness sign. One copy of this will be provided to the participant.

Conditions

Conditions: Diabetic Retinopathy

Keywords: Diabetes Mellitus
Diabetic Retinopathy
Referral

Study Design

Study Type: Interventional

Primary Purpose: Prevention

Study Phase: N/A

Interventional Study Model: Parallel Assignment

Number of Arms: 2

Masking: None (Open Label)

Allocation: Randomized

Enrollment: 180 [Anticipated]

Arms and Interventions

Arms	Assigned Interventions
Experimental: Health Education Intervention<br>Face-to-face health education session on Diabetic Retinopathy and available services at a tertiary hospital, followed by telephonic reminders at Day 7, 30 and 90.	Behavioral: Health Education Intervention<br>Face-to-face health education intervention on Diabetic Retinopathy and available services at a tertiary hospital, followed by telephonic reminder at Day 7, 30 and 90.

Arms	Assigned Interventions
No Intervention: Control Group<br>No Intervention will be conducted in this control group	

Outcome Measures

Primary Outcome Measure:

1. Increase in Successful Referrals
   Increase in Successful Referrals resulting from a health education intervention (among non-compliant persons, i.e. those who do not avail appointment with an Eye Consultant at a tertiary hospital after being detected with probable Diabetic Retinopathy at a diabetes hospital) - to be measured using Pre and Post intervention questionnaire based on previously used questionnaires of similar published studies

   [Time Frame: 5 months]

Secondary Outcome Measure:

2. Increase in knowledge about Diabetic Retinopathy
   Increase in knowledge about Diabetic Retinopathy (to be measured using Pre and Post intervention questionnaires based on previously used questionnaires of similar published studies; a total of 9 questions will be allotted to assess participants' knowledge on Diabetic Retinopathy. Accurate response rates regarding knowledge about Diabetic Retinopathy will be calculated by scoring responses out of 9. Participants who correctly answer atleast 7 will be considered to have good knowledge on Diabetic Retinopathy)

   [Time Frame: 5 months]

Eligibility

Minimum Age: 18 Years

Maximum Age:

Sex: All

Gender Based: No

Accepts Healthy Volunteers: No

Criteria: Inclusion Criteria:

- atleast 18 years of age
- Persons with diabetes who are registered with Barishal Diabetes Hospital
- Referred to SBMC&H (tertiary hospital) for DR management (screening/ treatment), from September 2017 – August 2018
- Persons who did not undergo a Dilated Fundus Examination (DFE) in last 12 months
- those who provide informed consent to be included in this study

Exclusion Criteria:

- Below 18 years of age
- Persons with diabetes who are registered with Barishal Diabetes Hospital but not referred to SBMC&H (tertiary hospital) for DR management
- Persons with diabetes referred to SBMC&H before September 2017 or after August 2018
- Persons who have undergone a Dilated Fundus Examination (DFE) in last 12 months
- those who do not provide informed consent to be included in this study

Contacts/Locations

Central Contact Person: Zara Khair, MDS
Telephone: 8801715342168
Email: zara.oikee@gmail.com

Central Contact Backup:

Study Officials: Michiko Moriyama, RN, MSN, PhD
Study Director
Hiroshima University

Locations:

IPDSharing

Plan to Share IPD: No

References

Citations:

Links:

Available IPD/Information:
