# Supplementary material for: Health education improves referral compliance of persons with probable Diabetic Retinopathy: A randomized controlled trial
Source: PLoS One. 2020 Nov 12;15(11):e0242047. doi: 10.1371/journal.pone.0242047 (PMC7660573; doi:10.1371/journal.pone.0242047)
Supplement: S8 File — (PDF) [file pone.0242047.s010.pdf]

ANNEXURE - C

FORMAT FOR SUBMISSION OF A RESEARCH PROPOSAL FOR ETHICAL APPROVAL

Project Title: Integrating Diabetic Retinopathy into Mainstream Health System in Bangladesh

SUMMARY:

Diabetes mellitus is a leading cause of death and disability.[1,2] Diabetic Retinopathy (DR) is an eye disease that affects the eyesight of about 21.3% patients with diabetes in Bangladesh in a way that is irreversible because of the nature of the disease.[18] According to International Diabetes Federation Diabetes Atlas (5th Edition), in 2011 there were approximately 8.4 million people in the age bracket 20-79 suffering from diabetes in Bangladesh, and this number is projected to double to 16.8 million by 2030. Based on these statistics, and also according to a nationally published report, the number of persons with DR is estimated to be about 1.8 million in Bangladesh.[10]

Taking measures to delay vision loss in patients with diabetes is more cost-effective than its treatment.[16] Timely and effective referral of DR cases to an Ophthalmologist is very important in the prevention of this disease.16 The objective of this study is to identify reasons of DR referral procedure compliance and non-compliance among registered diabetic patients in a diabetic clinic and recommend effective strategies to enhance the referral system.

The first phase of study is cross-sectional. Second phase of study is a Randomized Controlled Trial (intervention group to receive home-based health education and control group to receive standard care). The number of registered diabetic patients of Barishal DAB hospital who were referred to an Eye Consultant at a tertiary hospital following basic eye screening from September 2017 – August 2018 is N = 390 and they are all eligible for inclusion in the first phase of the study. This group (N = 390) will be divided into compliant and non-compliant patients. Both groups will be interviewed to understand motivation factors for compliance and de-motivating factors for non-compliance.

In the second phase of the study, the non-compliant group (N=300) will be divided further into intervention and control groups following computational Randomized Controlled Trial method. The intervention group will receive relevant health education messages on Diabetic Retinopathy and information about the days and times when eye care services are provided at the tertiary hospital (i.e. service availability information). They will be provided with telephonic reminders at Days 7, 30 and 90 after the health education. Then, after a gap of one month from the last telephonic reminder, both control and intervention groups will be interviewed again. In this way this study will conclude whether the health education session is an effective way of improving compliance rate of referred DR patients.

In-depth Interviews will be conducted with patients (compliant and non-

compliant) and service providers. All questionnaires will be pre-tested in the study location. All participants will read and sign a Bengali consent form where confidentiality issues, study procedures, modalities, and participants' right to refuse to answer/withdraw without monetary/non-monetary losses will be outlined in details. SPSS software will be used for data entry and analysis. Multiple Logistic Regression, along with other tests, will be used to identify variables that significantly influence successful referrals.

The outcome of this research will be shared with service providers managing diabetes as well as those providing eye care services in public and private sectors, academicians, scholars, experts, research institutions, policy makers and other stakeholders – with the aim to share reasons of compliance/non-compliance and recommend strategies to improve referrals. Ultimately, through this study trial, recommended strategies and best practices may be adopted, and replicated in other diabetic hospitals to integrate DR into mainstream health system effectively.

INTRODUCTION:

Diabetes is a non-communicable chronic disease associated with abnormally high levels of the glucose in the blood. One of the many complications of diabetes is Diabetic Retinopathy (DR), which results in vision problems in patients suffering from diabetes for many years.

DR is the fifth leading cause of global blindness, affecting an estimated 1.8 billion people and responsible for 4.8% of all causes of blindness.[1] According to International Diabetes Federation (IDF) Atlas, Bangladesh is a least developed country (LDC) with a disproportionately high proportion of people having diabetes. Among all people living with diabetes in the 48 LDCs, about 40% live in Bangladesh, which makes it one of the top 10 countries suffering from diabetes.[1] In Bangladesh, approximately 6.1% of the population aged 20-79 years has diabetes, with approximately 46% of these undiagnosed. According to International Diabetes Federation Diabetes Atlas (5th Edition), in 2011 there were approximately 8.4 million people in the age bracket 20-79 suffering from diabetes in Bangladesh, and this number is projected to double to 16.8 million by 2030. Diabetic Retinopathy (DR) and Diabetic Macular Edema (DME) are common complications of diabetes that can result in vision loss – the estimated number of people with DR in Bangladesh is about 1.8 million (21.3% of the diabetic patients).[18]

From a patient perspective there is little or no integration between the eye health sector and the diabetes sector. In Bangladesh, care for diabetes-related eye disease is available in very few tertiary level health facilities. There is currently no eye health care available for people with diabetes or those suffering from DR, at the community clinics, primary health care centers or secondary health care centers (The Fred Hollows Foundation, 2015).

An effective referral system ensures a close relationship between all levels of the health system and helps to ensure people receive the best possible care closest to home. It also assists in making cost-effective use of hospitals and primary health care services. A good referral system can help to ensure:

patients receive optimal care at the appropriate level, hospital facilities are used optimally and cost-effectively, patients who most need specialist services can access them in a timely way, primary health services are well utilized and their reputation is enhanced. A referral system at all levels is used as a means to facilitate flow of patient referrals among healthcare providers. It is an important activity in any healthcare system for it is a critical component of quality clinical care. If practiced efficiently, it can contribute to high standards of care by improving patient outcomes and decreasing costs through optimal use of medical services. An optimal referral process should be in place for the effectiveness, safety and efficiency of high standard medical care.

DR Screening is very important, because early stages of the disease lack symptoms – when symptoms occur the disease already requires treatment.[17] DR Screening centers may have to refer patients in a timely and effective manner to a DR treatment center. Early detection and timely referral are critical for timely interventions to prevent further deterioration of vision. In the case of DR, an effective referral system is crucial for the success of the disease management. This is because patient knowledge, awareness and perceived benefit of receiving DR Management services is very low, the world over, and more so in Bangladesh. Therefore, in order to make sure that DR Management is effectively integrated into mainstream public health in Bangladesh, it is vital to address one of the most challenging components of DR management, which is, strengthening referral system for patients having DR. If a patient is not made aware about possible consequences of negligence, is not properly counseled to undertake treatment and is not made aware of the existing referral pathway, the patient will in all probability forego treatment, since the perceived benefit of DR treatment is low among patients who lack symptoms.

<u>Bangladesh National strategy:</u> The Bangladesh National Eye Care Plan (2016-2020) has just recently incorporated DR management for the first time, indicating the government's commitment to manage DR through Public Private Partnership at all levels of the health system. [2] Bangladesh is also a signatory of Vision 2020, which is a global initiative for the elimination of avoidable blindness, a joint program of the World Health Organization (WHO) and the International Agency for the Prevention of Blindness (IAPB). Being a signatory of this initiative means that Bangladesh has pledged to work towards "A world in which no one is needlessly blind and where those with unavoidable vision loss can achieve their full potential". Given the DR disease burden in Bangladesh, effectively managing DR becomes important to fulfill these commitments. The prevalence of DR among people with diabetes is 27% in Bangladesh which is quite a concerning situation.

The Research Question is:
How can we increase the number of successful referrals of Diabetic Retinopathy patients from a diabetes clinic (DAB center) to an Eye Consultant located at a tertiary hospital?

OBJECTIVE:

General Objective: The general objective is to recommend health education messages regarding DR and intervention strategies to benefit people who are at risk of developing DR or has already developed DR.

Specific Objective: The specific objective is to investigate the effect of a health education intervention on patients with probable Diabetic Retinopathy who did not attend eye screening appointment with an Eye Consultant following referral from a diabetes clinic.

- <u>Primary Outcome:</u> Increase in Successful Referrals from 35% to 55% (among non-compliant patients, i.e. those who do not avail appointment with an Eye Consultant at a tertiary hospital after being detected with probable DR at a diabetic hospital)

- <u>Secondary Outcome:</u> Increase in knowledge of Diabetic Retinopathy and service availability (intervention vs. control groups)

RATIONALE:

A diabetic clinic should ideally be a one-stop-care center that offers holistic care for people with diabetes.[15] DR patients may be diagnosed or undiagnosed persons with diabetes. Before a person is screened or treated for DR, ideally that person must first be diagnosed with diabetes, and brought under comprehensive diabetes care management. Comprehensive diabetes care management includes management of diseases of organs including kidneys, heart, tooth, brain and eyes. Yet many undiagnosed persons with diabetes who suffer from DR may also walk into eye clinics, requiring urgent attention. In such cases, it is best to manage DR first and then refer the patient to a diabetes clinic, so that patient can be brought under comprehensive diabetes management. Controlling and managing diabetes will ensure that the DR condition of patient does not deteriorate. Management of diabetes ensures that DR is controlled or prevented. It is a well-established fact that prevention of DR is more cost-effective than its treatment. In order to delay the onset of DR, it is important that referred DR patients from Barisal DAB Center promptly uptakes services at the referred facility, i.e. the tertiary hospital. Referred patients should ideally avail advanced DR management and treatment options without further loss of time.

Diabetes clinics are one-stop-care-center for most registered patients with diabetes. Barisal DAB center is visited by thousands of diabetic patients each year. According to International Diabetes Federation, the national prevalence of DR among people with diabetes is 27%. It is estimated that 27% of these patients has Diabetic Retinopathy who are now able to undergo

basic eye screening for probable DR at the Barisal DAB. These are eye patients who are at risk of losing their vision. However, when they are referred to an Eye Consultant of a tertiary hospital for advanced DR screening and management, most than referred patients to not avail services in the tertiary hospital, due to lack of awareness, motivation and other barriers. These 'non-compliant' patients maybe assumed to 'drop out' of the health system (The Fred Hollows Foundation Bangladesh, 2018).

In the study locations:

	**Total Population**			**Adult (over 20 yrs) population**			**Probable number of persons with diabetes (6.1% of the total popn)**	**Probable number of persons with DR (27% of persons with diabetes)**
District	***Male***	***Female***	***Total***	***Male***	***Female***	***Total***		
Barisal Sadar	1,137,210	1,187,110	2,324,320	627,740	655,279	1,283,019	782,46	21,131

Sher-e-Bangla Medical College and Hospital (SBMC&H) has been providing eye care services for a long time to the community, and has recently started to provide eye care services to those suffering from Diabetic Retinopathy. Their services include eye screening and treatment for DR patients. The Eye Consultant in this hospital has received reputed international training. Most patients who came to seek DR management services at SBMC&H in 2017 and 2018 are registered diabetic patients of the Barishal DAB center who are referred from Barisal DAB center to SBMC&H (The Fred Hollows Foundation Bangladesh, 2018).

Barisal DAB center provides diabetes management services to registered diabetic patients, and has in recent year started to provide eye care services to those suffering from DR (The Fred Hollows Foundation Bangladesh, 2017). Their services include screening for probable DR cases. The medical personnel in this hospital has received training to screen for probable DR cases, and then refer these probable cases to an Eye Consultant for advance screening and management (preferably at SBMC&H since it provides DR treatment). From the patient record in the year 2017 and 2018, it has been found that most referred patients of Barishal DAB center do not avail their appointment at the referred facility (identified as non- compliant patients). These non-compliant patients are probable priority DR patients who need immediate medical attention (this risk group also has diabetes for more than 5 years, and/or a family history of diabetes, and/or a higher range of Body Mass Index along with worsening eye conditions) (The Fred Hollows Foundation Bangladesh, 2018).

Chances are these same diabetic patients (who have once before been referred from Barisal DAB center to an Eye doctor) will visit Barisal DAB center in future again for continued management of diabetes and other complication management. These high-risk patients can be followed-up with,

regarding their appointment at SBMC&H (The Fred Hollows Foundation Bangladesh, 2018). This is because timely referral is critical for DR patients, in order to delay further deterioration of their vision, and DR patients need to complete the referral cycle in a prompt manner. That is, the number of successful referrals (or completed referrals) is an important indicator.
From a patient perspective, the referral pathway should be clear and specific. Patients require specific information about the referred facility, such as days and times of services, how to travel to the referred facility, and so on. Through the health education component in this research, patients will be provided with this type of information, which will help them decide when and how to access tertiary level care. In this way, the non-compliance rate may be minimized.

This research will thus focus around the issue of 'DR patient referral management' in an in-depth manner, which has not been previously covered in any research in Bangladesh. This research will highlight health education messages and possible health education delivery strategies, targeting DR patients. This may then be incorporated and integrated into the existing health care delivery.

METHODOLOGY:

Study design: The following chart summarizes the study design. The first phase of study is cross-sectional. The second phase of study is a Randomized Controlled Trial (intervention group to receive home-based health education and control group to receive standard care).

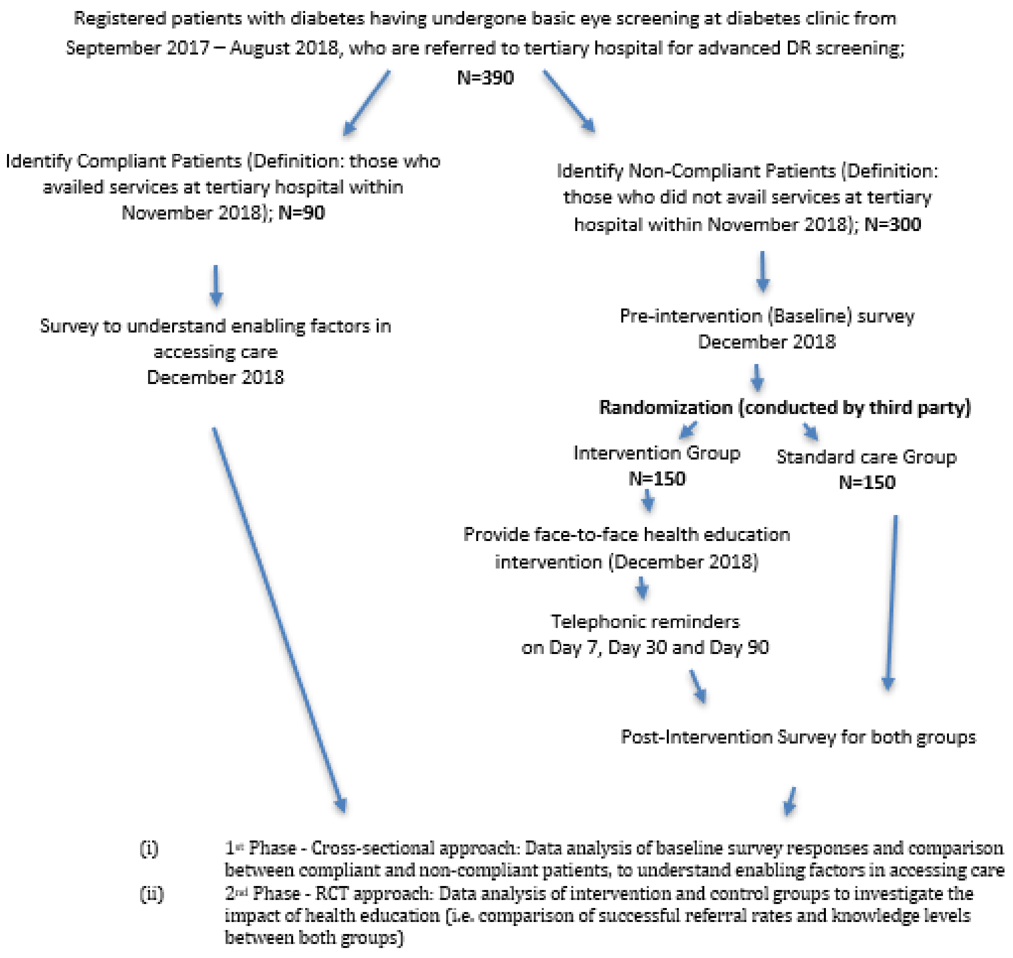

Figure 1: This flow-chart represents the study design

- Variables used in study: Education, Occupation, Income, Age, Sex, District of residence, DR Stage, time taken to travel to health facility, knowledge of DR, source of information on DR, accompanying person, decision maker, information received at diabetes clinic, counseling received at diabetes clinic, follow up/reminder calls about appointment with Eye Consultant, clarity about referral procedure (place/time/location/how to get there), satisfaction with health facilities, understanding of non-compliance on vision.

- Study Population: Registered diabetic patients who are/will be detected with probable DR and referred for specialty DR services at Sher-e-Bangla Medical College and Hospital (both groups will be studied – those who uptake services and those who do not, i.e. compliant and non-compliant patients)
- Inclusion criteria: All diabetic patients registered at Barisal DAB who are referred to SBMC&H for DR management (screening/treatment), following

basic eye screening, from September 2017 – August 2018 (i.e. during these 12 months) are eligible for inclusion in this study. Also, patients have to be atleast 18 years of age and will provide informed consent to be included in the research study. In addition to these criteria, in the 2nd phase of the study (RCT), only those patients will be included who did not undergo a Dilated Fundus Examination (DFE) in last 12 months.

- Exclusion criteria: Diabetic patients registered at Barisal DAB who are not referred to SBMC&H for DR management (screening/treatment), from September 2017 – August 2018 (i.e. during these 12 months) are excluded from this study. Also, patients below 18 years of age and those who do not provide informed consent to be included in the research study, are excluded from the study. In addition to these criteria, for the 2nd phase of the study (RCT), those patients who have undergone a Dilated Fundus Examination (DFE) in last 12 months will also be excluded from this study.
- Primary Outcome: Increase in Referral Rate (among non-compliant patients, i.e. those who do not avail appointment with an Eye Consultant at a tertiary hospital after being detected with probable DR at a diabetic hospital)
- Secondary Outcome: Increase in knowledge of Diabetic Retinopathy
- **Statistical basis of the sample size: We assumed that the intervention would result in an improvement of referral from 35% to 55% (i.e. 20% improvement). Thus to detect a difference of 20% in the referral with 90% power and type 1 error 0.05, the sample would be 125 in each group {sample size = [(p1xq1 + p2q2)/(p2-p1)^2] X factor for alpha and beta; where p1 is the percentage of the existing referral, q1 is 1-p1, p2 is the percentage of the expected referral from intervention; q2 is 1-p2, alpha is the type 1 error and beta if the type 2 error and factor for alpha beta with 90% power is 10.5}. Considering that for RCT study, single arm sample size is 125, this means both arm sample size = 125X2 = 250. Taking drop-out rate to be 20% (more than standard 10% because patients have already gone back home few months ago and it may be difficult to convince them to make the journey to the tertiary care hospital) the total sample size is 300 for the RCT study.**
- All diabetic patients registered at Barisal DAB who were referred to Barisal Sher-e-Bangla Medical College and Hospital after screening for probable DR (at the DAB clinic) from September 2017 – August 2018 are eligible for inclusion in this study. The total number of referred patients referred from September 2017 – August 2018 is N=390 based on hospital monthly data (from September 2017 – August 2018). All patients will be interviewed (both compliant and non-compliant patients).
- Recruitment strategy: All patients referred by DAB to a tertiary hospital from September 2017 – August 2018 following basic eye screening will be interviewed (both compliant and non-compliant patients), i.e. N=390. Both compliant and non-compliant patients will be studied. Interviews will be conducted to inform on the reasons for compliance and non-compliance.
- Methods of Data Collection: In-depth Interviews (IDI) with all patients. Patient phone numbers are recorded in hospital database. They will be contacted for consent for interview appointment and their detailed addresses will be noted for house visit. After completion of informed consent forms, face-to-face interviews with patients and Face-to-face interviews with service providers will be completed.
- Pre-testing: All the different sets of Questionnaires will be pre-tested in the study location which is Barisal district. The pre-test will be conducted on

both compliant and non-compliant patients. Feedback will be used to make any slight modifications, rearrangement of question sequence, changes in question wording or pattern, as and where needed. Since no sensitive questions are being asked to participants, it is expected that there will be no significant changes in the questionnaire that has been prepared.

- Data Interpretation: Responses to questions will be coded and entered into SPSS software, which will then be used to analyze and interpret data.
- Statistical Analysis: Frequency distributions will be used to analyze the demographic characteristics of the study population. Multiple Logistic Regression method will be used to identify variables that significantly influence successful and unsuccessful referrals among compliant and non-compliant patients. For the RCT study among non-compliant patients to find impact of health education intervention, the primary outcome (successful referral rate – defined as attendance at referred facility) and secondary outcome (increase in knowledge of Diabetic Retinopathy and service availability) will be compared between both arms. For continuous measures the t-test will be used and for categorical outcomes, chi-square test will be use, when comparing the two arms (i.e. intervention and control groups).

- **Utilization of Results:**
  The outcome of the research will be shared with service providers managing diabetes as well as eye care services, in public and private sectors, academicians, scholars, experts, research institutions, policymakers and other stakeholders. Researcher will arrange a dissemination seminar where government officials and INGO Forum members (working on eye health) will be present. Journalists will be invited to this event to publish findings in Bengali and English national dailies. She will present this research proposal, chapters from the Thesis as well as modified excerpts from the Thesis in various international and national platforms, conferences, symposiums and public health consortiums. Researcher will publish chapters from the thesis in peer-reviewed Public Health journals.

  Findings will help to strengthen the referral system of diabetic patients from DAB clinics to an Eye facility. This research will focus around the issue of 'patient referral' in DR management in an in-depth manner, which has not been previously covered in any research in Bangladesh to the best of researcher's knowledge. Therefore, strategies of strengthening the referral system of eye patients from DAB to an eye facility will be established.

- Facilities : (Resources, equipment, chemicals, subjects (human, animal) etc. required for the study):
  - Facilities Available : 1 computer, SPSS software, participants
  - Additional Facilities Required : None

Flow-chart

The following flow chart describes the sequence of tasks within the timeframe

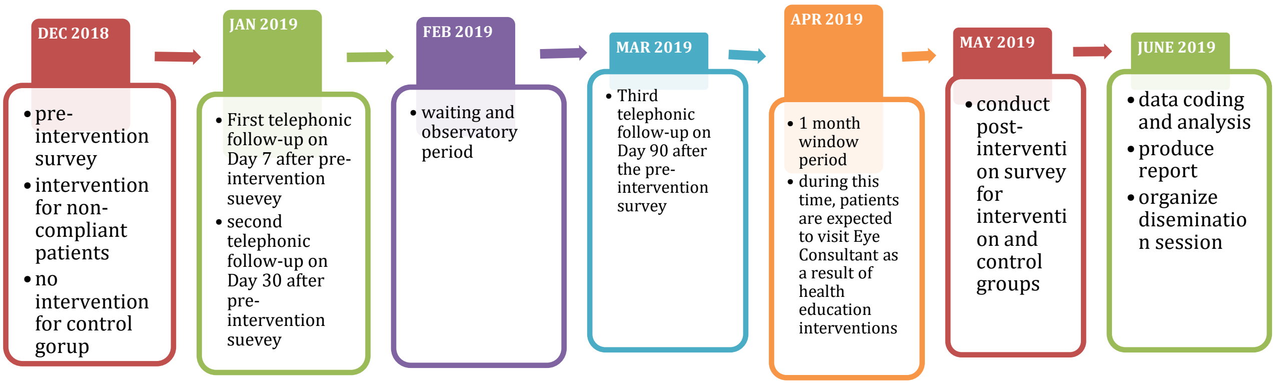

Ethical implications

There are no reasonable foreseeable (or expected) risks in this study as there will be no medical interventions. Participants will be made aware about the confidentiality issues, study procedures, modalities, and their rights to refuse to answer/withdraw at any point without any monetary/non-monetary loss.

This study is anonymous. Investigator will not be collecting or retaining any information about patient identity. The records of this study will be kept strictly confidential. Publications will not contain any information that may make it possible to identify participants.

Participants will not receive any monetary benefits for participating in this study. They will be informed about this before the start of the survey. They will be required to sign a detailed consent form that they can read in Bengali and have a witness sign. One copy of this will be provided to the participant.

References

1. Lozano R, Naghavi M, Foreman K, Lim S, Shibuya K, Aboyans V, et al., et al. Global and regional mortality from 235 causes of death for 20 age groups in 1990 and 2010: a systematic analysis for the Global Burden of Disease Study 2010. *Lancet* 2012; 380: 2095-128 http://dx.doi.org/10.1016/S0140-6736(12)61728-0 pmid: 23245604.
2. Murray CJ, Vos T, Lozano R, Naghavi M, Flaxman AD, Michaud C, et al., et al. Disability-adjusted life years (DALYs) for 291 diseases and injuries in 21 regions, 1990–2010: a systematic analysis for the Global Burden of Disease Study 2010. *Lancet* 2012; 380: 2197-223 http://dx.doi.org/10.1016/S0140-6736(12)61689-4pmid: 23245608.
3. IDF (2012) International Diabetes Atlas Fifth edition. Available: http://www.idf.org/atlasmap/atlasmap. Accessed 2018.
4. Bangladesh National Eye Care Plan, 2016-2020. Available: http://nec.gov.bd/opr_pdf/national_eye_care_plan.pdf
5. S.P. Mariotti, D. Pascolini, Br J Opthalmol. 2012, Global estimates of visual impairment: 2010
6. The Summary Report of The Bangladesh National Blindness & Low Vision Survey (30 & above age group), May 2003 (Childhood Blindness Study in Bangladesh)
7. The Bangladesh National Blindness and Low Vision Survey 2000
8. Ministry of Health and Family Welfare, Annual Report 2010-11
9. Erika Gebel, PhD, How Diabetes Differs for Men and Women, Diabetes Forecast, October 2011
10. https://www.dhakatribune.com/uncategorized/2015/09/16/idf-bangladesh-has-6m-diabetic-patients
11. S.P. Mariotti, D. Pascolini, Br J Opthalmol. 2012, Global estimates of visual impairment: 2010
12. The Fred Hollows Foundation Bangladesh. (2015). *Barisal Situational Analysis*. Internal report: unpublished.
13. The Fred Hollows Foundation Bangladesh. (2017). Internal report: unpublished.
14. The Fred Hollows Foundation Bangladesh. (2018). Internal report: unpublished.
15. Lawton, J. , Parry, O. , Peel, E. and Douglas, M. (2005), Diabetes service provision: a qualitative study of newly diagnosed Type 2 diabetes patients' experiences and views. Diabetic Medicine, 22: 1246-1251. doi:10.1111/j.1464-5491.2005.01619.x
16. Javitt JC, Aiello LP. Cost-effectiveness of detecting and treating diabetic retinopathy. Ann Intern Med. 1996;124(1:Pt 2):t-9.
17. Singer DE, Nathan DM, Fogel HA, Schachat AP: Screening for diabetic retinopathy. Ann Intern Med 116:660–671, 1992
18. Akhter A , Fatema K , Ahmed SF , et al . Prevalence and associated risk indicators of retinopathy in a rural Bangladeshi population with and without diabetes. Ophthalmic Epidemiol 2013;20:220–7

Budget:

The total budget for this study is BDT forty-five thousand only (Tk 45,000/). For detailed breakdown, please refer to Appendix E.
